# Psychosocial work environment in Swedish primary healthcare: a cross-sectional survey of physicians’ job satisfaction, turnover intention, social support, leadership climate and change fatigue

**DOI:** 10.1186/s12960-024-00955-4

**Published:** 2024-10-23

**Authors:** Hanna Fernemark, Nadine Karlsson, Janna Skagerström, Ida Seing, Elin Karlsson, Emma Brulin, Per Nilsen

**Affiliations:** 1 Department of Health, Medicine and Caring Sciences, Division of Health and Society, Linköping University, 581 83 Linköping, Sweden; 2 Primary Health Care Centre, Lambohov, Region Östergötland Sweden; 3 Research and Development Unit, Region Östergötland, Linköping, Sweden; 4 Department of Behavioral Science and Learning, Linköping University, 581 83 Linköping, Sweden; 5 Unit of Occupational Medicine, Institute of Environmental Medicine, Karolinska Institutet, Stockholm, Sweden; 6 School of Health and Welfare, Halmstad University, Halmstad, Sweden

**Keywords:** Psychosocial work environment, Change fatigue, Primary healthcare, Physicians, Discontent with current job

## Abstract

**Background:**

Primary healthcare, the first line of care in many countries, treats patients with diverse health problems. High workload, time pressure, poor job control and negative interpersonal experiences with supervisors have been documented in primary healthcare. The work environment in primary healthcare is also affected by several types of changes.

**Aim:**

We aimed to explore the levels of job satisfaction, turnover intention, social support, leadership climate and change fatigue according to physicians in Swedish primary healthcare. We also aimed to identify and characterize physicians exhibiting both high turnover intention and low job satisfaction, i.e., “discontent with current job”.

**Methods:**

A cross-sectional survey based on a random sample of physicians working in Swedish primary healthcare.

**Results:**

Approximately one-quarter of the respondents were discontented with their current job. Discontent was negatively associated with poor general health and change fatigue among the respondents; social support from colleagues and a favorable leadership climate showed positive associations in terms of reducing the levels of discontent with current job.

**Conclusions:**

The findings of this study highlight the association between low levels of job satisfaction and high levels of turnover intention (i.e., discontent with current job) among physicians in primary healthcare. Moreover, these variables exhibited a strong association with physicians’ general health; poor health significantly increased the likelihood of discontent with current job. Our findings also show that experiencing change fatigue is associated with discontent with current job among physicians in primary healthcare. This knowledge can help identify and improve shortcomings within the psychosocial work environment in Swedish primary healthcare.

## Background

The demand for primary healthcare has increased significantly due to numerous factors, including aging populations and an increasing number of people living with one or more chronic conditions [1–3]. Studies on primary healthcare have documented that high workload, time pressure, poor job control, and negative interpersonal experiences with supervisors [4, 5] lead to poor job satisfaction and high turnover intention among physicians and other healthcare workers in this setting [6, 7].

Research in Sweden and other western societies has associated poor working conditions in primary healthcare with work-related health problems, such as poor self-rated mental health, well-being and high levels of job dissatisfaction [7, 8]. Adverse working conditions for primary healthcare physicians in Sweden have led to a debate about the risk of a shortage of physicians in future [9–13]. Turnover among physicians is associated with increased organizational costs, reducing the resources available for patient care [14]. Turnover intention has been shown to predict actual turnover among physicians [15, 16]. As the number of physicians decreases, the burden on those who remain will increase, leading to higher likelihood of burnout, sickness absence and staff turnover, resulting in a detrimental cycle. To gain a better understanding of what might contribute to staff shortages in primary care, this study aimed to explore the characteristics of physicians in primary healthcare exhibiting both high levels of turnover intention and low levels of job satisfaction by combining these measures into the concept of “discontent with the current job”. By identifying and analyzing both personal characteristics and work-related factors of physicians experiencing job discontent, we can create targeted strategies to prevent burnout and promote overall employee work-related health and well-being.

Beyond job dissatisfaction and high turnover intention, earlier research has identified several other detrimental factors that negatively affect the psychosocial work environment in healthcare, such as high workload, moral distress, and frequent organizational changes [19–21]. It is well known that frequent organizational changes serve as stressors and may result in reduced levels of employee well-being, decreased job satisfaction, and increased turnover [8, 22–24]. The work environment in primary healthcare can be affected by changes, such as epidemiological shifts, digitalization and technical development, medical breakthroughs [25], new clinical guidelines and organizational changes [25–29].

Work-related changes are necessary for the development of primary healthcare, but they can contribute to stress and poor working conditions [30]. The COVID-19 pandemic forced changes in primary healthcare workers’ daily routines and environment. They had to deal with immediate, sometimes poorly prepared, organizational changes and routines, which negatively affected their working conditions and work-related health [31, 32]. Research has shown job dissatisfaction may be a result of frequent changes in the primary care organization, necessitating changes in work roles [33]. Rapid and continuous changes can also cause change fatigue, defined as an overwhelming feeling of stress and exhaustion associated with workplace changes [34]. An instrument developed by Bernerth et al. [34] to measure change fatigue includes six items to investigate employees’ perceived change fatigue. Earlier research using this measure showed that change fatigue among hospital nurses had a negative influence on their job satisfaction but a positive influence on resilience, i.e., the ability to thrive in the face of adversity [26]. We have not identified any other studies examining the association between change fatigue and discontent with work.

On the other hand, social support is suggested as a factor contributing to retention and job satisfaction among physicians [33, 35]. Social support [37, p. 69] is defined as “the overall levels of helpful social interaction available on the job from co-workers and supervisors”. Research has shown that social support from colleagues can protect against adverse health outcomes related to psychological stressors at work and is important for long-term well-being and stimulation of productivity at work [36]. Leadership is another factor that may contribute to physician retention and job satisfaction [35]. A favorable leadership climate, defined as “the emotional tone and mutual trust set by leaders within the work environment” [37], has been shown to be associated with job satisfaction among nurses [38]. Studies of employees other than healthcare personnel have shown that leadership climate can positively affect the empowerment and performance of individuals and work groups [37].

This study addresses several knowledge gaps relevant to understanding physicians’ working conditions in primary healthcare and developing solutions for improvement. To our knowledge, the extent to which primary healthcare physicians in Sweden experience high turnover intention and low job satisfaction and their association with change fatigue, social support and leadership climate has never been investigated. This knowledge is important to gain insights into mechanisms that could potentially reduce physicians’ turnover intention and increase their job satisfaction, which could contribute in the long term to the creation of a more sustainable, attractive work environment for recruiting and retaining physicians in Swedish primary healthcare.

## Methods

### Setting

The survey was conducted in Sweden, where healthcare is the responsibility of 21 county councils. All citizens are insured by the state to ensure equal access to healthcare and fees are standardized and regulated by law [39, 40]. Primary healthcare is the first line of care in Sweden and treats patients without limitations regarding disease, age, or certain groups of patients. Primary healthcare in Sweden thus has a gatekeeping function for secondary care, requiring patients to obtain a referral from the primary healthcare unit, except in the case of emergencies, where patients can seek care at the emergency room for acute illnesses at any time [41]. Primary healthcare is responsible for actions in terms of medical judgment, treatment and preventive work as well as rehabilitation that does not require specialized care. Primary healthcare also has the medical responsibility of care homes for the elderly [40].

### Study design, sample and procedure

The study is a cross-sectional survey based on a stratified, random sample of physicians in Swedish primary healthcare. Data were drawn from the 2022 Longitudinal Occupational Health Survey in Healthcare Sweden (LOHHCS) study [43]. The LOHHCS study was initiated in 2021 and included a representative sample (*n* = 6699) of physicians in the occupational registers. It is an open cohort, meaning that at follow-up, an additional sample was drawn from those newly registered in the occupational register and those with a medical degree in the national educational register (UREG). The educational register was used because of its immediate update compared with the occupational register, which has a 1-year delay in the update. In 2022, 7908 physicians were invited to participate; of these, 2712 answered the questionnaire (34% response rate). We were interested in the working conditions for physicians in primary healthcare. Thus, those respondents who stated that they worked in primary healthcare (*n* = 1099) were selected for inclusion in this study.

The survey was distributed to the participants between March and May 2022. One invitation and three reminders were sent. The respondents were informed that they could answer the questionnaire online or by filling out the paper form sent with the invitation letter. Statistics Sweden (SCB) was responsible for power calculations, sampling, distributing the questionnaires and gathering the data. Partial missing data for the sample varied between 0.0 and 2.5% for most questions. Statistics Sweden is responsible for official statistics and other government statistics and develops and produces statistics for use in research [44].

### Questionnaire

The LOHHCS questionnaire was divided into four sections: (A) medical and professional background included title, workplace, clinical experience, part- or full-time work and turnover intention; (B) work environment included several instruments measuring different aspects of the work environment, for example, job satisfaction and leadership climate; (C) health included questions regarding, for example, general health; (D) background demographics, such as marital status and number of children living at home.

### Measures

The measures included in this study were as follows: discontent with the current job (the combined measure of low job satisfaction and high turnover intention); social support from colleagues; leadership climate; general health; change fatigue. Data on gender and age were retrieved from Statistics Sweden’s registers. The remaining background variables were retrieved from the questionnaire: clinical experience; country of education; having a partner or not; having children living at home or not; full-time or part-time job; main employer; average weekly work hours; number of days on sick leave in the last 12 months.

### Discontent with current jo

We constructed an outcome measure, “discontent with current job”, to identify and study the characteristics of respondents expressing low job satisfaction and high turnover intention. The rationale for this combination was the idea that respondents expressing high turnover intention and low job satisfaction would be more discontent with work and, hence, potentially be at higher risk of, for example, work-related ill-health. To isolate factors specifically related to turnover, we wanted to eliminate variables that might influence a physician’s decision to their current position unrelated to job dissatisfaction, such as relocating for family reasons or receiving alternative job offers. This ensures that the study accurately attributes turnover to low job satisfaction rather than external factors. The measure was created by combining the responses to the questions concerning job satisfaction and turnover intention. Those who responded “quite unsatisfied” or “very unsatisfied” to the job satisfaction item and also responded regarding turnover intention that they were thinking about leaving the job “every day”, “a couple of times a week” or “a couple of times a month” were considered to exhibit “discontent with the current job”.

### Job satisfaction

Job satisfaction has been described previously as the extent to which a person reports overall contentment with various aspects of their work, such as career opportunities, incentives and salary [45, 46]. Job satisfaction was measured with one question: “How satisfied or unsatisfied are you with your job?” and response options varied from “very satisfied” to “very dissatisfied”.

### Turnover intention

Turnover intention can be defined as an individual’s willingness to voluntarily leave their current job [47]. Turnover intention was measured with one validated [47] question: “How often during the last 12 months have you considered quitting your job?” and response options ranged from “every day” to “never”.

### Social support from colleagues

Social support from colleagues was measured with two items from Copenhagen psychosocial questionnaire, earlier validated elsewhere [48]: “Item 28b: If you need, you will get help and support from your colleagues?” and “Item 28c: If you need to, you can talk to your colleagues about situations with patients or relatives who have been particularly difficult?” with response options on a Likert scale ranging from 1 (always) to 5 (never) [48]. Cronbach alpha for social support from colleagues was 0.82. We reversed the scale for the analysis; therefore, a high value on the scale for social support from colleagues corresponds to a good level of social support.

### Leadership climate

Leadership climate was measured with a validated 10-item instrument [49, 50] with statements, such as “I am clear about what my boss expects of me” and “My boss encourages my participation in the set-up of my work” with response options ranging from “yes, often” to “no, never”. Participants could also choose “not relevant” for this measure. Cronbach alpha for leadership climate was 0.90. We reversed the scale for the analysis, and therefore, a high value on the leadership climate variable corresponds to a good leadership climate.

### Change fatigue

Change fatigue was measured using the change fatigue measurement scale, which is a validated instrument containing six items with a Likert scale [34]. The scale has shown good reliability and internal consistency in larger sample populations, including recent use in a nursing population [26, 51]. The scale was originally developed to explore the impact of multiple organizational changes on employee well‐being, organizational commitment and turnover intention, including items such as “I am tired of all the changes in this organization” [34]. The original change fatigue scale uses a 7‐point Likert scale ranging from 1 (strongly disagree) to 7 (strongly agree) to determine the level of perceived change fatigue. In our study, we used a 5-point Likert scale on the advice of SCB after scrutiny of the questionnaire; the responses were as follows: (1) strongly disagree, (2) disagree, (3) neutral, (4) agree, (5) strongly agree. Cronbach's alpha for change fatigue was 0.91, indicating the scale's internal consistency.

Earlier research has shown that individuals may interpret questions in ways other than intended. Therefore, a pre-test inspired by cognitive interviewing [52] was conducted. HF and JS tested the translated items linguistically and semantically on four physicians and two nurses, respectively. The participants were asked to answer the translated version of the instrument by expressing their thoughts and interpretations of the questions out loud with either HF or JS present in the room, taking notes and asking questions when needed for clarification. JS and HF discussed the findings together with PN and IS, which resulted in minor revisions and clarifications of a few terms in some of the questions.

The change fatigue instrument was translated into Swedish by the authors for use in the present study. The translation was subsequently reviewed by bilingual colleagues to ensure accuracy.

### General health

General health was measured with the validated question [48], “In general, how do you perceive your health?” with Likert scale response options ranging from 1 (excellent) to 5 (bad).

### Statistical methods

When items in a scale were missing, a total score was calculated if at least half of the questions were answered, and the missing items were given the average score of the other items in the scale [53]. The reliability of the psychosocial scales (leadership climate, social support from colleagues and change fatigue) was estimated with Cronbach α internal consistency coefficient. The distribution of the sample characteristics and discontent with the current job was estimated for all study respondents. Descriptive statistics are presented as frequencies or mean values and standard deviation (SD).

Logistic regression was used to identify the characteristics of those who had high discontent with their current job. The analysis was unadjusted in model I and multivariables were adjusted in model II for gender, age, clinical experience, overtime work, leadership climate, social support from colleagues, change fatigue and health. Odds ratios (ORs) for high levels of discontent with the current job were estimated with 95% confidence intervals (CIs). The psychosocial scales (leadership climate, social support from colleagues and change fatigue) used as independent variables in the logistic regression were standardized (mean, 0; SD, 1 for the purpose of comparability.

Results were considered statistically significant at *p* < 0.05 using two-tailed tests. Statistical analyses were performed with SPSS28.

## Results

### Respondent characteristics

The response rate for the physicians was 34% (2712 of 7908). Demographics are presented in Table 1.

**Table 1:** Characteristics and results for the survey respondents among physicians in primary healthcare

Study variables	Number	Mean ± standard deviation	Frequency (%)
Age	1099	45.8 ± 12.1	
≤ 29 years			74 (6.7)
30–39 years			330 (30.0)
40–49 years			314 (28.6)
50–59 years			182 (16.6)
≥ 60 years			199 (18.1)
Gender	1099	–	
Male			459 (41.8)
Female			640 (58.2)
Country of education	1098	–	
Sweden			868 (79.1)
Other European country			159 (14.5)
Other			71 (6.5)
Having a partner	1095	–	
Yes			990 (90.4)
No			105 (9.6)
Children at home	1079		
Yes			636 (58.9)
No			443 (41.1)
Working full-time or part-time	1099		
Full-time			571 (52.0)
Part-time			528 (48.0)
Clinical experience	1097		
< 5 year			189 (17.2)
5–10 years			234 (21.3)
10–15 years			232 (21.1)
> 15 years			442 (40.3)
Work hours	1096		
≤ 40 h			553 (50.5)
> 40 h			543 (49.5)
Overtime work (at least once a month in the last 12 months)	1096		
Yes			925 (84.4)
No			171 (15.6)
Leadership climate	1066	30.6 ± 6.3	
Social support from colleagues	1079	8.7 ± 1.6	
Change fatigue	1092	16.2 ± 6.1	
General Health	1097		
1 Excellent			160 (14.6)
2 Very good			385 (35.1)
3 Good			327 (29.8)
4 Quite good			174 (15.9)
5 Poor			51 (4.6)
Sickness absence (in the last 12 months)	1085		
None			940 (86.6)
1–5 days			78 (7.2)
> 5 days			67 (6.2)

The mean value for leadership climate indicated that the respondents perceived their leadership climate as relatively favorable. Similarly, the mean value for social support from colleagues suggested strong levels of perceived support among the respondents. The average level of change fatigue indicated that participating primary care physicians were fatigued slightly below the “neutral” level.

Descriptive results for job satisfaction, turnover intention and the constructed discontent with the current job variable are presented in Table 2, showing that 25% of the respondents were discontent with the current job.

**Table 2:** Descriptive statistics of the primary outcome of “discontent with current job” among physicians

Study variables	Number	Frequency (%)
Job satisfaction	1098	
1 Very satisfied		202 (18.4)
2 Quite satisfied		529 (48.2)
3 Neither satisfied nor unsatisfied		192 (17.5)
4 Quite unsatisfied		134 (12.2)
5 Very unsatisfied		41 (3.7)
Turnover intention (How often during the last 12 months have you considered getting a new job)	1098	
1 Every day		78 (7.1)
2 Once/a couple of times a week		130 (11.8)
3 Once/a couple of times a month		208 (18.9)
4 Once/a couple of times in the last 12 months		339 (30.9)
5 Never		343 (31.2)
Discontent with current job	1097	
No		828 (75.5)
Yes		269 (24.5)

### Regression analyses

The crude analysis showed no significant difference between male and female physicians regarding discontent with their current job (Table 3). Clinical experience of 10–15 years had a strong association with discontent with the current job in crude analysis. Change fatigue, poor health and overtime work were also associated with significantly increased levels of discontent with the current job. A favorable leadership climate and an adequate social support from colleagues demonstrated decreased levels of discontent with the current job in the crude model.

**Table 3:** Logistic regression analysis of discontent with current job among physicians in primary healthcare

Variables	Model I (crude)^a^			Model II (multivariable)^a^		
	OR	95% CI	*p* value	OR	95% CI	*p* value
Gender						
Male	1			1		
Female	1.07	0.81–1.42	0.638	0.71	0.51–1.01	0.056
Age (continuous)	0.98	0.97–0.99	< 0.001	0.96	0.94–0.99	0.006
Clinical experience						
< 5 years	1			1		
5–10 years	1.46	0.94–2.28	0.092	2.16	1.26–3.72	0.005
10–15 years	1.64	1.06–2.55	0.028	2.82	1.52–5.23	< .001
> 15 years	0.83	0.54–1.25	0.369	1.88	0.87–4.07	0.108
Overtime work						
No	1			1		
Yes	2.77	1.70–4.52	< 0.001	1.50	0.85–2.64	0.161
Social support from colleagues	0.52	0.45–0.59	< 0.001	0.69	0.58–0.82	< 0.001
Leadership climate	0.38	0.32–0.44	< 0.001	0.47	0.39–0.56	< 0.001
General health						
Excellent	1		< 0.001	1		
Very good	2.40	1.25–4.58	0.008	2.02	0.99–4.09	0.052
Good	4.82	2.55–9.10	< 0.001	3.17	1.58–6.39	0.001
Quite good	8.05	4.15–15.61	< 0.001	5.77	2.77–12.04	< 0.001
Poor	22.46	9.87–51.11	< 0.001	11.62	4.40–30.69	< 0.001
Change fatigue	1.59	1.37–1.84	< 0.001	1.41	1.18–1.67	< 0.001

OR: odds ratios; CI: confidence interval

^a^Model I is crude. In model II, ORs are adjusted for all other variables in the table (gender, age, clinical experience, overtime work, leadership climate, social support from colleagues, change fatigue, and general health)

In the multivariable analysis, there were no significant gender differences in relation to discontent with the current job. In contrast, age showed significant differences in the crude and the multivariable models, revealing that the levels of discontent with the current job decreased as participants’ age increased. Discontent with the current job was almost three times more likely among respondents with 10–15 years of clinical experience compared with those with less than 5 years of experience. In the multivariable analysis, physicians with 5–10 years of clinical experience were twice as likely to be discontent with their current job compared with those with clinical experience of < 5 years. However, the results show that discontent with the current job when having more than 15 years of clinical experience was not statistically different compared to those with less than 5 years of experience.

Working overtime was significantly associated with discontent with the current job in the crude analysis, but when controlling for other variables, this significance did not remain. In the crude and multivariable analyses, respondents who reported poor health indicated a significantly higher level of discontent with their current job. Participants who reported good health had significantly higher levels of job discontent than those with excellent health.

In the multivariable model, a favorable leadership climate had a significant association with reduced levels of discontent with current job. Feeling supported by colleagues also had a significant association with reduced levels of discontent with current job and experiencing change fatigue had an association with increased levels of discontent with current job.

## Discussion

This study showed that one in five respondents was neither satisfied nor unsatisfied with their job and a similar proportion reported thinking about quitting their job once or a couple of times a month. Change fatigue was experienced to some extent, and leadership climate and social support from colleagues were rated quite highly among the respondents.

We found that nearly one-fourth of the respondents were discontent with their current job, which aligns well with earlier studies. Previous research has shown that general practitioners (GPs) in the United Kingdom perceive their work as unsustainable, with many considering early retirement as a possible solution [30] and have expressed concerns regarding the present and future lack of physicians wanting to work in primary healthcare due to the workload [19, 30]. This is also relevant in Sweden [54].

Working overtime could be viewed as an aspect of high workload, which has been identified as a source of job dissatisfaction among primary healthcare physicians in many Western countries [55]. However, overtime work was not significant in the multivariable analysis in this study and earlier research comparing physicians in primary healthcare showed higher levels of job dissatisfaction among Swedish physicians, even though a larger proportion of physicians in Norway reported regularly working > 50 h per week [55]. Physician job dissatisfaction is not only devastating for personal health and well-being but is also a risk factor for the quality and outcomes of medical care [6, 8]. Low job satisfaction among physicians could potentially act as a barrier to the recruitment and retention of physicians in primary healthcare. Research has shown that dissatisfaction with work among GPs in the United Kingdom is associated with attrition [56].

Our results showed that almost one-third of the respondents thought about leaving their current job from several times a week to a couple of times a month. Research has shown that intention to leave one’s job predicts actual turnover [15]. Moral distress among physicians and nurses has also been shown to affect turnover intention [57]. Degen et al. [18] found that drivers for turnover intention among physicians were poor psychosocial working conditions and work-related pressure when working with patients. Associations between intention to leave primary healthcare and burnout symptoms among physicians have also been reported [58].

One in four respondents in our study was discontent with their current job. Research has shown that experiencing a low level of job satisfaction is a strong predictor of turnover [59, 60]. The poor psychosocial working conditions in primary healthcare are well known and the prevalence of burnout is high among physicians in Sweden [7, 31, 43, 61, 62]. Our findings are, therefore, a cause for concern because even respondents who perceived their health as fairly good were three times more likely to be discontent with their current job compared with those with excellent health. Turnover not only affects physicians and their workplaces but is also a costly burden on the healthcare system [14, 63]. The issue with turnover in healthcare is not exclusive to physicians. Similar results are found in other professions within the healthcare sector [64]. We found that physicians who have 10–15 years of clinical experience were more likely to be discontent with current job. This group of physicians is middle-aged and consists of foremost residents. Burnout and poor job satisfaction have previously been found to be the highest among physicians aged 31–50 years [65].

Organizational research has shown that to implement changes successfully, employees need to feel prepared for and value the change [67]. Changes initiated by the professionals themselves have been found to be more feasible to implement and changes that employees are unprepared for or do not fully understand tend to be difficult to implement [29]. Healthcare is continuously exposed to various changes [20, 30]. Therefore, supervisors and employers must communicate and facilitate understanding of how changes will affect employees. Periods of stability, when no organizational changes occur, have been found to be important for employees’ well-being [20, 34, 67, 68].

Among other professions in healthcare, experiencing change fatigue has been shown to be associated with lower levels of job satisfaction and be significantly associated with changes in depression and stress levels [26, 69]. However, research on this topic is limited, particularly among physicians. Considering the relevance of change fatigue for many work environment issues, more research is warranted.

The findings concerning low job satisfaction and high turnover intention suggest that primary healthcare in Sweden may face difficulties retaining physicians in future. The Swedish National Health Competence Council estimates a shortage of approximately 2200 primary healthcare physicians in 2035; therefore, the number of physicians needs to double in Sweden to meet the needs of the population [54]. Sweden has one of the lowest physician-to-population ratios among comparable Western countries [70], with 0.62 physicians in primary healthcare per 1000 population; the Netherlands and Canada have 0.9 and 1.33 physicians per 1000 population, respectively. This is problematic because primary healthcare in Sweden is expected to take on the role of developing an integrated care model to achieve more holistic care to prevent diseases, manage chronic conditions, bring care closer to home and educate the population on better self-care [70]. With high turnover rates of physicians in primary healthcare in Sweden exacerbating the existing shortage, the situation has become increasingly challenging. The National Board of Health and Welfare has outlined a target of one physician for every 1100 patients in Swedish primary healthcare [71]. However, our research reveals that nearly a quarter of physicians are dissatisfied with their current job, making it difficult to attain this goal.

Despite uncovering negative aspects of the psychosocial work environment of physicians in Swedish primary healthcare, we also identified protective aspects that mitigate job discontent. A favorable leadership climate and feeling socially supported by colleagues corroborate earlier studies on protective psychosocial factors at work [36, 72]. Social support from colleagues and supervisors in a workplace is an important predictor of increasing intention to stay in the organization and improving job satisfaction [73, 74]. In a Swedish context, research suggests that empowering leadership decreases the turnover intent among primary healthcare physicians in Sweden [75]. Because the Swedish healthcare system is governed by political decisions [40] and influenced by different stakeholders, such as the Swedish Association of Local Authorities and Regions [76], we believe that our findings are important regarding the protective factors.

By combining low job satisfaction with high turnover intentions, we aimed to create a stronger predictor for staff turnover. The findings in our study are also important regarding minimizing discontent with the current job among physicians in primary healthcare. Employers need to identify employees with poor health because our results indicate that a considerable number of physicians in Swedish primary healthcare are experiencing inadequate levels of health, which in turn was associated with being more discontent with their current job. While it is difficult to ascertain whether individuals with poor general health view their job situation more negatively or if a negative job situation contributes to declining health, we consider this discovery important and warrant further investigation. Previous studies have indicated that factors like the work environment have a greater impact on burnout and stress levels compared to gender and age, underscoring the importance of delving deeper into this relationship [77]. Understanding this relationship is crucial to developing appropriate actions to improve job satisfaction and reduce turnover, e.g., by decreasing workload and time spent on unnecessary work tasks [56, 73].

### Methodological considerations

This study has some methodological considerations that need to be addressed. We used a quantitative research method to investigate a population of physicians at one point in time, which made a cross-sectional survey appropriate. This type of research makes it possible to reach many individuals in a short time and at low cost. However, it does not allow for causal inferences because cross-sectional studies can only point to statistical associations between variables [78]. Another limitation was the relatively low response rate (34%), restricting the generalizability of the findings. Comparison with the target population showed that there were relatively more physicians born in Nordic countries or with a Swedish background among the survey respondents. As with much research, there is a risk that participants are primarily those who have a strong interest in the topic or are most able to respond. Previous studies, such as Brodie et al. [80], have shown that individuals who are more motivated and opinionated are more likely to complete surveys [79]. Additionally, factors like lack of motivation, heavy workloads, poor timing and incorrect addresses can lead to dropouts and non-responses [80]. Consequently, it is reasonable to assume that the survey respondents in our study had either particularly favorable or unfavorable attitudes toward the issues being studied.

Research has established that motivated and opinionated people are more likely to respond to surveys [78]. Thus, those who did respond to the survey may have had more favorable or more unfavorable attitudes concerning the issues under study. The study also has considerable strengths. The study is a large cross-sectional survey of physicians employed in primary healthcare. Furthermore, both individual and work-related factors were investigated. The method allowed us to identify patterns at a certain time that would be of interest to employers, management and politicians.

## Conclusion

Almost a quarter of physicians in Swedish primary healthcare are discontent with their current job, exhibiting low job satisfaction and high turnover intention. Physicians with poor general health were more discontent than physicians with excellent health. Experiencing change fatigue increases levels of discontent with their current job among physicians in primary healthcare. A favorable leadership climate and a supportive social environment among colleagues are associated with reduced levels of discontent with their current job among physicians in Swedish primary healthcare.

## Data Availability

Ethical laws prevent the free sharing of data.
